# Situation analysis on the integration of refractive error services provided by optometrists into the national health services in Kenya

**DOI:** 10.1186/s12889-024-18960-6

**Published:** 2024-05-29

**Authors:** Shadrack Muma, Kovin Shunmugam Naidoo, Rekha Hansraj

**Affiliations:** 1 College of Health Sciences, Department of Optometry, University of KwaZulu-Natal, Durban, South Africa; 2 ONESIGHT EssilorLuxottica Foundation, Paris, France; 3 PO Box 811, Kisumu, Kenya

**Keywords:** Refractive error, Refractive services, Kenya, Eye care, National health service

## Abstract

**Introduction:**

Even though the burden of uncorrected refractive error could potentially be addressed through innovative and cost-effective approaches, integration of the services into the National Health Services (NHS) is desirable. However, minimal information exists on the current situation warranting the need for evidence about the integration of refractive error service provided by optometrists into the national health services in Kenya.

**Methods:**

A situation analysis of the Kenyan refractive error services provided by optometrists within the NHS was undertaken based on access to service delivery, service coverage, and human resource. A strengths, weaknesses, opportunities, and threats analysis was undertaken based on the existent evidence to identify the core factors that could potentially facilitate or hinder the integration of refractive error services provided by optometrists within the National Health Services. The proportion of optometrists to be integrated in the NHS was estimated based on the minimum ratios recommended by the World Health Organization.

**Results:**

A section of tertiary and secondary healthcare facilities in Kenya have specific services to address refractive errors within the NHS with most facilities lacking such services. Treatment of refractive error occurs at the level of eye care general services. There are 11,547 health facilities offering primary care services in Kenya. However, none of them offers refractive error services and only a section of facilities offering county health referral services provides eye care services which is limited to refraction without provision of spectacles. The existing workforce comprises of ophthalmologists, optometrists and ophthalmic clinical officers, together with nurses and other general paramedical assistants. Optometrists, ophthalmologists and ophthalmic clinical officers are allowed to undertake refraction. However, optometrists majorly practices in the private sector. Centralization of eye care services in urban areas, weak referral systems, and a shortage in the workforce per population was observed.

**Conclusions:**

The Kenyan NHS should advocate for primary care and reorient the current hospital-based delivery approach for refractive error services. This is attributed to the fact that provision of refractive error services at primary care remains effective and efficient and could translate to early detection of other ocular conditions. The existing human resources in the eye health ecosystem in Kenya should maximize their efforts towards addressing uncorrected refractive error and optometrists should be integrated into the NHS.

## Introduction

Globally, uncorrected refractive error (URE) is the leading cause of visual impairment that could be addressed by a pair of spectacles [1]. While the prevalence of URE is estimated at 6.39% in Kenya, there is a dire need for population based studies to provide an accurate prevalence to allow for action and allocation of resources [2]. Notwithstanding, considering that majority of the underserved population are in need of refractive error (RE) services in developing countries such as Kenya, there is a need to frame RE services within universal health coverage. Despite URE being the leading cause of vision impairment globally, there is lack of empirical data on vision impairment due to URE in Kenya to allow for proper health planning warranting the need to consider the global burden of this condition [3]. Considering the demographic trends around age, the increase in the prevalence of myopia and high myopia globally, and other chronic eye diseases over the coming decades, innovative approaches for service delivery are desirable to address the population’s needs [4]. Therefore, integration of RE services provided by optometrists into the National Health Services (NHS) would address the principles of universal health coverage. The integration concept is majorly geared towards enhancing accessibility and scaling RE services through promotive, preventive, treatment, rehabilitation, and palliative health services needed, when and where they are needed, and without financial constraints. Accessibility of health services can be divided into physical accessibility, financial affordability and acceptability [5–7].

Achievement of universal health coverage demands that health services should be physically accessible, financially affordable, and acceptable to the public. In countries such as the UK, the concept of primary eye care has received recognition and optometrists are managing chronic eye conditions through simple interventions to prevent people from resourcing to the hospitals. The primary eye care in developed countries such as the UK’s NHS, is currently provided by optometrists, general practitioners, and ophthalmic medical practitioners [8]. Over recent years in Kenya, Masinde Muliro University, Kenya Medical Training College and Kaimosi Friends University introduced optometry programs with an aim of scaling human resources to deliver eye care services including URE in Kenya [9]. Even though approximately 400 optometrists have been trained, they are not integrated into the public health sectors and majority operates within the private independent optical sectors which are majorly located in urban areas [10]. However, given that 72% of Kenyans resides in rural areas [11], they are not able to access the RE services provided by the optometrists. Notably, within the Kenyan NHS there are no specific services to address RE within the public health sectors warranting the need for optometrists integration [12]. Normally, the management and treatment of RE occur at most tertiary level healthcare facilities with most RE patients in need of spectacles being referred to the private sectors [12]. This highlights the need for integration of RE services provided by optometrists within the NHS so that the population in need can access the services across all levels of healthcare delivery for the achievement of universal health coverage.

In Kenya, most of the RE services are delivered in the private sector, which includes the private medical sector and the optical sector with a weak referral pathway and minimal integration within the NHS [13]. Notwithstanding, eye care services available within the Kenyan NHS are fully hospital based and are almost entirely at the tertiary care level with a few at secondary care level with provision of RE services limited to refraction without availability of spectacles [12]. The Ministry of Health currently advocates for screening for ocular conditions such as RE at the primary care level but the approach remains weak due to the lack of adequate human resources [14]. This implies that there is absence of eye care services at the primary care level with the existing services limited to screening without comprehensive refraction services provided by optometrists. Therefore, there is a need to adopt a methodology incorporating innovative approaches inclined towards improving the system as a whole with stringent primary eye care [15]. To the best knowledge of the authors, this is the first situation analysis of RE services provided by optometrists in Kenya, and the integration of the services into the NHS, present in the literature. This study will act as a baseline for research and to monitor the commitment of Kenya towards recognizing the burden of URE and the need for integrating RE services provided by optometrists into the NHS so as to achieve effective RE coverage by 2030 [16].

## Materials and methods

A situation analysis of the Kenyan NHS capacity to address refractive error was conducted. The keywords used were as follows: national OR eye health OR refraction OR national strategic plan AND Kenya. This review was intended to understand the government of Kenya efforts towards addressing URE and the recommendations in the strategic plans. The World Health Organization (WHO) Eye Care Situation Analysis Tool (ECSAT), which supports countries in the planning and evaluation of eye care services, served as a basis for that analysis [17]. The information for the situation analysis was collected from accessible and official sources from the Ministry of Health, such as national reports and planning documents and workforce inventory frameworks. Official organization databases were systematically searched to retrieve all potentially relevant reports/documents about refractive error services in Kenya. The national strategic plans for eye health review were downloaded and thereafter reviewed systematically with a focus on the recommendations on addressing URE from the first strategic plan and the achievements in the subsequent strategic plan. The scope of practice for ophthalmic workers in Kenya was also retrieved from the Ministry of Health website for review. The review was intended to identify the roles assigned to each eye care professional category in Kenya. The scope of practice for ophthalmic workers in Kenya was also retrieved from the Ministry of Health website for review. The review was intended to identify the roles assigned to each eye care professional category in Kenya. The intention of the review was to provide a justification for the need of integrating RE services provided by optometrists into the public healthcare sector in Kenya. The information was divided according to ECSAT components. The ECSAT components were informed by, and categorized under the six WHO health system-building blocks [18]. Three of those building blocks were used to conduct this study:

1. Accessibility service delivery: number of NHS centres providing RE services and waiting times for an eye care/refractive care assessment.
2. Service coverage: distribution of the available services by geography and population density.
3. Human resources: number of eye care professionals in the NHS centres providing RE services. These three building blocks were selected based on the nature of the services to be assessed. The objective of this study was to give a comprehensive overview of the availability of refractive error services in the country’s NHS [19].

A strengths, weaknesses, opportunities, and threats (SWOT) analysis was performed by the three authors based on the existent evidence to identify the core factors that can potentially facilitate or hinder the possible integration of RE services provided by optometrists within the NHS in Kenya. The initial elements for the SWOT analysis were derived from a systematic review. Each author presented their views regarding the SWOT elements derived from the review before a consensus was reached. A systematic search was conducted in multiple national and international electronic scientific databases, such as MEDLINE/PubMed, Web of Science, Scopus, and Google Scholar to retrieve all relevant publications about RE services. A comprehensive search strategy was conducted combining terms related to the eye condition (refractive error, myopia, hyperopia, presbyopia) and terms related to the outcome of interest (refractive error services, primary eye care, eye care services). No time interval for the study’s conduction has been defined. For every publication or paper found, the reference list was reviewed searching for additional studies or data in an attempt to retrieve all the relevant information. The rationale for the review was to obtain an objective SWOT of the Ministry of Health actions towards addressing URE in Kenya. Based on the reviews, the authors adopted open ended questions from a study by Zoschke et al. [20] as shown in Table 1. Thereafter, the authors crosschecked the SWOT analysis from the review to ascertain if they answer the open ended questions. The authors reached a consensus after discussing the elements derived from the review for the SWOT analysis and the questions. Information was extracted and categorized according to the SWOT domains of strengths, weaknesses, opportunities and threats.

1. Strengths: positive impacts and effects in the health system and population health status.
2. Weaknesses: disadvantages and negative effects in the health system and population health status.
3. Opportunities: elements that can be used in advantage to improve the health system and population health status.
4. Threats: elements that can cause disadvantages and compromise the health system and population health status.

**Table 1:** The SWOT analysis questions

**Strengths**:
1. What are the strengths of the policy documents and the national strategic plans for eye health towards addressing URE?
2. What are the strengths of policy documents and the national strategic plans towards achieving RE integration into the NHS?
**Weaknesses**:
1. What are the weaknesses of the policy documents and the national strategic plans towards integration of RE into the NHS?
**Opportunities**:
1. What good opportunities are available for integration into policy documents and the national strategic plans to help in achieving RE integration into the NHS?
2. What are the new and exciting trends that policy documents and the national strategic plans can try to help in achieving RE integration into the NHS?
**Threats**:
1. What problems do the policy documents and the national strategic plans face which limits them from achieving RE integration into the NHS?
2. Could any of the weaknesses within the policy documents and the national strategic plans threaten RE integration into the NHS?

Priority areas to be addressed were defined based on the situation analysis, including human resources. For human resources planning, the number of eye care professionals to integrate was calculated considering the minimum ratio recommended per population by the WHO: one optometrist per 50,000 populations and one ophthalmologist per 15,000 populations [21].

## Results

**Fig. 1:**
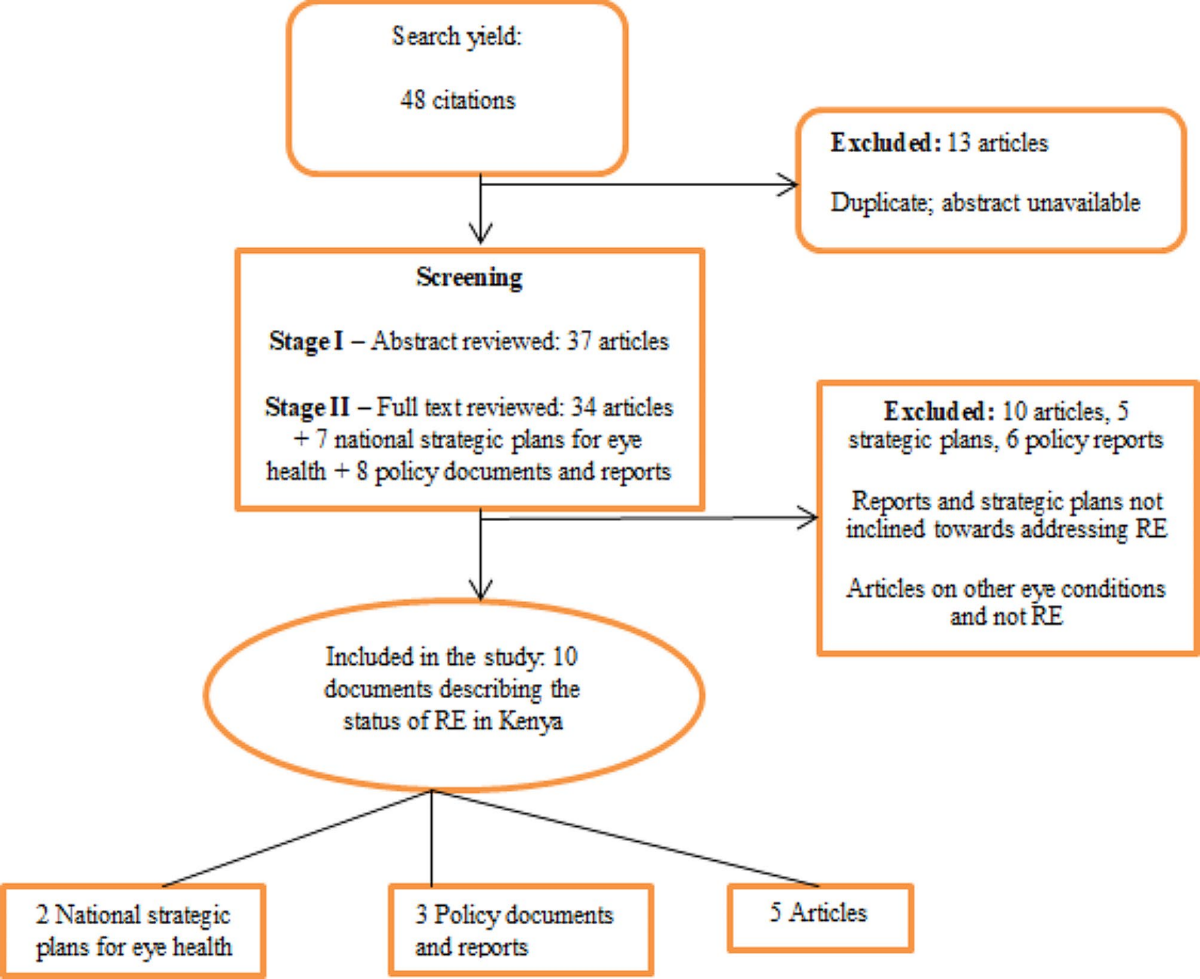
Review search strategy

Figure 1 illustrate the number and type of documents which yielded the results for the SWOT analysis.

### Current capacity of the Kenyan NHS to address refractive error

The NHS eye care services in Kenya are hospital-based and divided into two categories, majorly the human resources capacity and the differentiation of cadre. Category I eye care services are provided by ophthalmologists, ophthalmic clinical officers and ophthalmic nurses who are operating within the public health sectors and offers services to approximately 100,000 people. The training and competency for Category I eye care professionals is shown in Table 2.

**Table 2:** Training and competency for Category I eye care professionals in Kenya [22]

Cadre	Duration of Training	Scope of Practice
Ophthalmologists	3–4 Years after a degree in Medicine and Surgery	Medical and Surgical management of eye conditions or management with optical devices
Ophthalmic clinical officers	2 Years after a diploma in clinical medicine	Comprehensive clinical Ophthalmic Care
Ophthalmic Nurse	1 Year after a degree or diploma in nursing	Ward and Clinic based nursing care and management

The category I services majorly includes the general assessment of eye conditions for pathology and management, surgical interventions for conditions such as cataract and refraction which has not adequately been addressed by this category. Category I eye services provides a wide range of eye services but have resources in terms of integration into the NHS and human resources when compared to category II [22]. Category II eye services are provided by optometrists, optical technicians and ophthalmic opticians who majorly operates within the private sectors and offers services to approximately 250,000 people. The training and competency for Category II eye care professionals is shown in Table 3.

**Table 3:** Training and competency for Category II eye care professionals in Kenya [22]

Cadre	Duration of Training	Scope of Practice
Optometrists	4–5 Years	Comprehensive vision and Optical care, management of restricted range of eye rehabilitation
Ophthalmic opticians	3 Years	Dispensing of Optical Prescriptions
Optical technicians	1 Year	Cut and fabricate Lenses onto the spectacles

The category II services are majorly refraction which is undertaken within urban areas. According to the scope of practice for ophthalmic workers in Kenya, category I has approximately 603 eye care professionals while category II has approximately 400 [22]. In Kenya at the NHS level, the ophthalmic clinical officers, ophthalmologists, ophthalmic nurses and other ophthalmic paramedical assistants constitute the integrated workforce [22]. In Kenya there are three categories of health facilities services namely primary care services, county health referral services and national referral services [23]. Primary care services are offered in 11,547 facilities, county health referral services are offered in 839 facilities and national referral services are offered in 7 facilities [23]. However, out of the 47 counties in Kenya, only Bomet County, Uasin Gishu County and Nairobi City County have integrated category II eye care professionals into the public health sectors at the county health referral services. Notwithstanding, such integration have been limited to just one county health referral services while facilities offering primary care services do not offer refractive error services.

Based on the geographical distribution of NHS eye care services in Kenya, the centralization and coverage of services is fundamental. Majority of sectors providing RE services are mainly in urban areas. Data from the Ministry of Health Kenya shows that all the 47 counties have a referral healthcare facility offering eye care services [12]. Out of the 47 county referral healthcare facilities offering eye care services, only 3 offers comprehensive RE services including availability of spectacles [12]. Again given that all of the 47 county referral healthcare facilities are mostly located within urban areas, the populations in need of RE services living in rural areas are underserved. Notwithstanding, with a population of approximately 47,564,294 Kenyans, approximately 951 optometrists are required if the WHO recommendation of one optometrist per 50,000 population is to be adopted [11]. Ideally there is a deficit of 551 optometrists in Kenya considering that the existing optometrists in Kenya are approximately 400 [12]. There are approximately 151 ophthalmologists in Kenya according to the Ministry of Health [12]. Therefore, if the recommendation of one ophthalmologist per 15,000 population is to be adopted then a deficit of 3,019 ophthalmologists still exists in Kenya [12]. While there are no recommended ratios for allied ophthalmic personnel emphasizing the need for such recommendations with an aim of scaling human resources to undertake refraction. Even though in the absence of one category of eye care professionals, the other cadres could address the condition and lack of all of them is a key barrier when it comes to accessibility of refractive error services.

In Kenya, RE services are not provided as a differentiated care and a proper and functional referral system resulting to an extensive waiting list for a general eye care assessment hence compromising a timely delivery of care. The situation does not only apply to RE but for other conditions that are placed at the same list of priority. Between the years 2018 and 2019, referrals from primary care to ophthalmology increased from 108,234 to 121,867, an indication for a demand for eye care services. Of those, 74,345 − 91,712 were left unattended, respectively, with the median waiting time increasing from 65 days to 93 days, with a maximum of 104 waiting days for an eye care assessment [12]. More recent data from 2022 shows that 73% of the hospitals providing eye care services do not meet the recommended response times when it comes to addressing RE [12].

While integrating the NHS RE services at the secondary care level is considered ineffective and expensive considering the limited eye care resources, the Kenya NHS has various shortcomings [24]. However, the delay in integration of optometrists into the primary level presents a missed opportunity that could be considered to ensure that referrals are made when necessary. This will potentially reduce the long waiting time and early detection at the primary levels.

## Current refractive error services available in Kenya

While the aspect of out-of-pocket payments for RE services has been a key barrier to the underserved population, patients who manage to check their RE status ends up being unable to access the prescribed optical devices. With challenges around the NHS in which RE services are not integrated, patents are forced to seek the services at the private medical sector and optical shops at an increased fee. Apart from the exposure to financial risk, the lack of specific regulation that defines the roles of the person who dispense, the training requirements and medical devices dispensing regulation exposes patients to considerable risks to public health [25]. The government-approved training of optometrists to undertake refraction in public universities and colleges in Kenya was started in 2009 at Masinde Muliro University and 2006 at Kenya Medical Training College [26]. However, given that the Ministry of Education, Science and Technology and the Ministry of Health operates independently, the Ministry of Health does not consider the optometrists as a regulated health workforce.

Even though there is formal academic training, optometry in Kenya remains the only eye care discipline which is unregulated with no legislative framework that ensures guidelines for practice [14]. In consideration that most RE services in Kenya are undertaken in the private sector and optical shops, the absence of specific policies and regulations regulating the dominant sectors exposes the population in need of the services to benefit at a considerable financial burden.

## Problem analysis and setting priorities (SWOT analysis)

The SWOT analysis from the review showed that RE services are delivered on an independent basis, with minimal integration of RE services provided by optometrists within the NHS [27]. Notwithstanding, out of the seven national strategic plans for eye health in Kenya only the strategic plans for the period 2012–2018 and 2020–2025 mentions RE.

A substantial primary care based eye care covering differentiated and multidisciplinary care integrated within the NHS and across other sectors has been shown through empirical evidence and socio-economic analysis as ideal for addressing eye care [15]. Although there are challenges around health systems reforms and health [12], the care, and refractive services specific solutions more holistic for Kenya is to explore the available human resources specifically the optometrists and the existing infrastructures in the country and implement policy and regulatory changes to address this condition.

Considering the global burden of URE and the impact on the quality of life when the correction in most cases requires a simple pair of spectacle [28], actions should be directed towards scaling innovative interventions towards provision of services. In addition, the interventions of screening of vision and eye conditions will potentially address timely detection and referral, hence enhancing health gains for the patient and efficiency gains for the health service [29].

The need for RE services is anticipated to increase which translate to an increase in the prevalence and population demographic variations. Therefore, it is fundamental to prepare eye care professionals to rapidly respond to the expected demand. The strengths, weaknesses, opportunities, and threats – SWOT analysis was undertaken as shown in Table 4. Based on the existing evidence [12], internal and external factors that could potentially facilitate or hinder the possible integration of RE services within the NHS were identified. Actions to be made to implement refractive services within NHS according to relevant WHO health systems building blocks are summarized in Table 5.

**Table 4:** SWOT analysis of the integration of refractive error services at primary care level within the Kenyan NHS, based on the existent evidence [22]

**Strengths**	**Weaknesses**
• Presence of eye care professionals at the county referral hospitals who can do refraction.	• Lack of refractive error services such as spectacles within the public health sector.
• Integration is cost-effective.	• Task shifting integrated with telemedicine.
• Timely management of other ocular conditions such as allergies.	• Restructure the referral system.
• Integrate refractive error services into primary care to enhance accessibility.	
**Opportunities**	**Threats**
• Establish a proper referral pathway to allow timely referral for eye care services.	• Lack of a structured regulatory scope of practice and competency framework for eye care workforce in Kenya.
• Scale eye care services to healthcare facilities situated within rural areas.	• Paradigm shift
• Integrate refractive error services to the public health sector.	

**Table 5:** Summary of actions to implement refractive services at primary care within the NHS

Governance	Workforce and infrastructures	Financing	Service delivery
Establish policies and regulations inclined towards addressing refractive error services.	• Regulate and integrate optometrists into the primary eye care workforce.	• Integrate refractive error services into the public health sectors.	• Prioritize primary care to scale accessibility and refractive error coverage.
Integrate refractive error services into the existing primary care infrastructures	• Implement the National Strategic Plans for eye health.	• Integrate refractive error services into the National Hospital Insurance Fund.	• Integrate refractive error into the NHS to address affordability barriers.

The core problems and barriers which were identified in the access to RE services within the NHS were categorized into three domains as follows:

1. Lack of primary eye care that could potentially address RE at the primary care level, in relation to proximity to the population.
2. Physical barriers to accessing the services with centralization of eye care services in urban healthcare facilities with a weak referral pathway.
3. Shortage of human resources limiting accessibility to eye care services.

## Discussion

Addressing URE demands proper integration across the relevant health programs targeting the health of the general public like the child health. Application of a public health perspective should be utilized in addressing URE given the potential of the approach in enhancing equity in the access, quality of the services, and effective coverage. Considering that interventions to address URE are desirable, the interventions should be integrated within the NHS given its significance to the population [30]. Evidence shows that integrating RE services into primary health care, potentially scale accessibility to the community [31]. Therefore, to ensure universal access to RE care, with health and financial protection, this study provides recommendation from scientific evidence, technical recommendations, and experiences gathered from other countries. The key consideration is integration of refractive services provided by optometrists within the NHS primary care network.

Evidence has shown that addressing RE services in short-term programs and in areas lacking regulation potentially compromises the sustainability and service delivery [32]. While addressing RE remains essential given the high prevalence among the population, an integrated intervention is worthy of attention. Patients with RE should be assessed periodically and services should not only be quality but should also be accessible. Again cost-effective practices are worth implementing, prioritizing adoption of innovative interventions are desirable to protects public health, patients, and professionals who provides care where is needed, when is needed without exposing the user to financial barriers. Therefore, integration of RE services into the NHS could create a stable provision of services across various healthcare levels.

Primary health care is defined as “the first level of contact of individuals, the family and community with the national health system bringing health care as close as possible to where people live and work, and constitutes the first element of a continuing health care process” [15]. The aspects around promotion, prevention, and treatment of RE are categorized within this level of care and the integration into the NHS grants on the sustainability and service delivery for long-term provision of services.

Social determinants of health are sustainable service delivery in RE and should be considered in improving the population health status. To ensure a sustainable RE service delivery, integration into the primary care centres should have the following characteristics [33, 34]:

1. Eye care should be comprehensive and not only provides refractive services but should also entail detection and screening of other ocular conditions. The aspect of referral, awareness creation and community engagement should be strengthened.
2. Enhance accessibility of RE services through creation of vision centres within the primary level to address barriers around travelling cost.
3. Scale coverage of RE services across different geographical locations regardless of the point prevalence.
4. Enhance continuity of RE services through integration within the NHS.
5. Enhance quality of RE services which should be effective, safe, and timely and people centred. Policies should be prioritized to ensure an effective and safety of services.
6. Prioritizing a people centred RE services based on an individual acceptance and responsive and not on financing basis.
7. A strengthened referral pathway between different levels is desirable to ensure a well-functioning refractive service.
8. Striving towards enhancing an accountable and efficient RE services without wastage of resources.

Addressing the Kenyan population’s refractive needs and to respond to the human resources shortage within the NHS, integration of a new professional eye care category such as the optometrists within the NHS to work alongside the existing ophthalmologists is desirable. Globally, optometry has been the main provider of RE services, Kenya included from the private sector setting [14]. The population of trained optometry professionals in Kenya is approximately 400 in 2022, according to data from the Ministry of Health, ophthalmic service unit, with the minimum academic qualifications of a Diploma [12].

Utilization of existing optometrists in Kenya by the NHS is desirable given that the populations are in need of RE services. However, to achieve the integration, regulations and establishment of policies to recognize integration of optometrists and their roles in RE service delivery is justifiable. Integrating optometrists within the primary care will ensure effective RE service delivery and detection of other ocular conditions hence timely referral to other levels of care or medical specialties such as ophthalmologists [12]. Despite the training of optometrists from the government institutions in Kenya, a proper mechanism hasn’t established to integrate optometrists into the public health sectors [35]. Even though some counties in Kenya have integrated optometrists into the public health service with a monthly remuneration of approximately US$ 300 which is lower than the private sector rates of US$ 500, most optometrists within the private sector still fights for integration into the public health sectors [35]. This is attributed to lack of job security within the private sectors and optical sectors. Hence, the private sectors and the optical sectors take advantage of the optometrists trained from the government academic institutions for purposes of profit generation with minimal focus on social impact [12]. Therefore, the public sectors should facilitate integration of optometrists into the public sector to ensure budget savings for the government and utilization of the resources invested in training of the workforce. In developed countries such as UK, evidence shows that optometrists are effective when it comes to provision of primary eye care. Even though there are fewer ophthalmologists in the UK, suitable training and accreditation of optometrists has been shown as a safe and effective approach in the management of not only RE but also other chronic eye conditions [8].

Looking at RE from a health-economics point of view, adoption of cost effective and innovative intervention would potentially address more than 90% of unmet eye care needs [36]. Again integration of RE services within the NHS could also address financial barrier which limits accessibility to RE services by majority [36]. While globally, URE impacts negatively on the economy with annual global productivity losses from uncorrected myopia in adults and presbyopia estimated at USD 244 billion and USD 25.4 billion, respectively, integration of RE services into the NHS is desirable [37]. Given the economic burden attributed to URE, a strong health economic rationale for scaling RE services into the primary care in combination with other public health interventions are desirable [38]. In addition, it has been shown that optimization of vision functional ability could potentially results to employment, enhanced work productivity, increased household income, and enhanced economic productivity of not only individuals but to the nation. This should not only apply for people with full visual potential but also for individuals with vision impairment [39].

Therefore, given the current economic, sustainability of the NHS and the financial situation in the country, actions towards a strengthened primary care is imperative. The primary- centred health systems have been shown to be relatively cost effective, responsive to health care needs and ensure equity in access [40].

Given the endorsement of the global targets for eye care and the effective RE coverage by the WHO [16, 41], implementation of refractive services in the Kenyan NHS is desirable. In conclusion, the Kenyan NHS should integrate RE services provided by optometrists into the primary care. The human resources available currently in the country have the potential to scale RE services if cadres such as optometrists who dominantly operate within the private health sectors could be integrated into the public health sectors. The provision of refractive services at primary care should be prioritized as it has been shown to be efficient and effective and could potentially scale recognition of other eye conditions.

## Data Availability

Data is provided within the manuscript.

## Abbreviations

NHS: National Health Service
RE: Refractive Error
URE: Uncorrected Refractive Error
